# Circadian and Genetic Modulation of Visually-Guided Navigation in *Drosophila* Larvae

**DOI:** 10.1038/s41598-020-59614-y

**Published:** 2020-02-17

**Authors:** Ece Z. Asirim, Tim-Henning Humberg, G. Larisa Maier, Simon G. Sprecher

**Affiliations:** Department of Biology, Institute of Zoology, University of Fribourg, Fribourg, Switzerland

**Keywords:** Neuroscience, Circadian rhythms and sleep

## Abstract

Organisms possess an endogenous molecular clock which enables them to adapt to environmental rhythms and to synchronize their metabolism and behavior accordingly. Circadian rhythms govern daily oscillations in numerous physiological processes, and the underlying molecular components have been extensively described from fruit flies to mammals. *Drosophila* larvae have relatively simple nervous system compared to their adult counterparts, yet they both share a homologous molecular clock with mammals, governed by interlocking transcriptional feedback loops with highly conserved constituents. Larvae exhibit a robust light avoidance behavior, presumably enabling them to avoid predators and desiccation, and DNA-damage by exposure to ultraviolet light, hence are crucial for survival. Circadian rhythm has been shown to alter light-dark preference, however it remains unclear how distinct behavioral strategies are modulated by circadian time. To address this question, we investigate the larval visual navigation at different time-points of the day employing a computer-based tracking system, which allows detailed evaluation of distinct navigation strategies. Our results show that due to circadian modulation specific to light information processing, larvae avoid light most efficiently at dawn, and a functioning clock mechanism at both molecular and neuro-signaling level is necessary to conduct this modulation.

## Introduction

Circadian rhythms are ≅ 24 h oscillations displayed by various organisms^1–4^, maintained by an endogenous timekeeping mechanism that can be entrained to the environment by external cues called ‘zeitgebers’ (German for “time-givers”) such as light, temperature and even social interactions in mammals^5–7^. Through entrainment, organisms anticipate and importantly, adapt to daily environmental oscillations^8,9^ in order to regulate physiological phenomena and behaviors associated with locomotion, sleep patterns, hormone release and body temperature among others^10–15^. In the absence of entraining stimuli, circadian rhythmicity is self-sustained in a “free-running” state^2,16^. Molecular components responsible for the organismal ability to sustain rhythmicity have been extensively characterized and the pacemaker mechanisms display conserved patterns between fruit flies and humans^17,18^. Given that many metabolic processes are highly correlated with circadian clocks, the disruption of circadian rhythms may lead to abnormal behavioral rhythms, altered mood, depression, sleep disorders in humans^19–23^ and it has been reported to impact type 2 diabetes and cancer^24–26^. Likewise, the ability to properly synchronize endogenous clocks with circadian time was shown to positively impact fitness in fruit flies and various other organisms^27–29^.

The molecular clock mechanism in *Drosophila* is composed of interlocking transcriptional feedback loops^30^ presenting homologous components with the molecular circadian mechanism in mammals^18^. In one loop, CLOCK (CLK) and CYCLE (CYC) proteins heterodimerize (CLCK/CYC) in the cytoplasm and translocate to the nucleus where they positively regulate the expression of *period* (*per*) and *timeless* (*tim*) genes^31–34^. Protein products of these genes (PER and TIM) accumulate in the cytoplasm during late day/early evening and heterodimerize to translocate to the nucleus later in the evening^35–39^. Here, PER represses the activity of CLK/CYC, hence negatively regulating its own transcription as well as *tim* transcription^38,40^. TIM is bound by Cryptochrome (CRY) in the nucleus, which is the key and the only circadian-dedicated photoreceptor in *Drosophila*^41^ responsible for promoting light-dependent degradation of TIM as a molecular response to light in order to reset the molecular clock^42–45^. Eventually, before dawn, both PER and TIM are degraded, releasing CLK/CYC to resume their activity^46^. The rhythmic activity of core molecular clock components characterizes the circadian pacemaker circuitry. While the molecular clock is conserved developmentally, the fruit fly larva displays a relatively simpler neuronal organization than its adult counterpart, and particularly, an accessible clock network consisting of nine neurons per brain hemisphere, thus representing an attractive model to study circadian-dedicated neural circuits. Specifically, larval pacemaker neurons comprise neuropeptide pigment-dispersing factor (PDF)-expressing lateral neurons (PDF-LaNs) as the main pacemaker neurons, a 5^th^ PDF-negative lateral neuron (5^th^-LaN) and two sets of dorsal neurons (DN1 and DN2)^47,48^.

Owing to the experimental advantages and considerable homologies with mammals, *Drosophila* has been extensively used as a model system to study the genetic and cellular mechanisms as well as the fundamental neural circuits of circadian rhythmicity and entrainment of the molecular clock^49^. The main behavioral outputs tackled in *Drosophila* circadian rhythm studies are locomotor activity and eclosion, since robust rhythms can be observed and recorded in individuals or fly populations^50,51^. Although these studies are conducted by using adult flies, also larvae have been used as a circadian rhythm model^52–55^. Besides being equipped with a comparable neuronal simplicity, fruit fly larvae also exhibit well-characterized attraction/avoidance behaviors in response to environmental cues such as temperature, chemicals and light^56–58^. Moreover, in response to sensory stimuli, larvae are able to make compound decisions, by modulating two alternate moto-programs as runs and turns in order to navigate towards a preferred condition or away from an undesirable stimulus^59–61^. As extensively described for photo-navigation of innately photophobic larvae, they employ distinct navigation strategies which are defined by the length, size, direction and frequency of runs and turns, operated by processing of spatial or temporal cues^62–64^.

**Figure 1:**
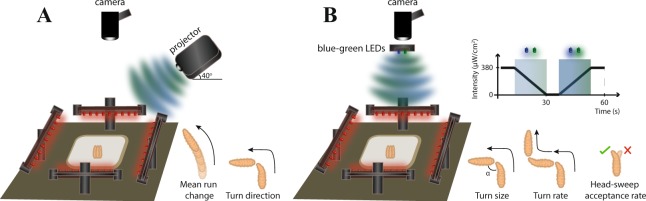
Schematic representation of the experimental setup. 30 larvae were placed in the middle of the behavioral plate illuminated by red LEDs. Larval navigation was recorded by a camera placed above. Two distinct setups were used in order to measure spatial and temporal navigation strategies which were both designed to present light stimulation within the overall range of spectral sensitivity of both larval photoreceptor subtypes. (**A)** A directional light gradient is created by placing the projector as a light stimulus source from one side of the behavioral plate. Navigation strategies measured through this setup are termed as spatial navigation strategies, defined with respect to the light source and consist of *mean run change*; the degree of steering within a run, and *turn direction*; the percentage of turns biased away from the light source. (**B)** A temporal light gradient is introduced by blue and green LEDs with cycling light intensity. The light intensity linearly decreases and increases between 380 and 0 μW/cm^2^ for 25.5 s, interspaced by constant light intensity phases for 4.5 s. 1 cycle of temporal intensity change is completed in 60 s. Navigation strategies measured through temporal light information processing are *turn size*; the degree of turn angle, *turn rate*; the average number of turns an animal makes per minute, and *head-sweep acceptance rate*; the percentage of accepted head-sweeps, termed as temporal navigation strategies. Constant light intensity phases were not taken into consideration during data analysis. Only larval behavior during linear light intensity increase and decrease was taken into account for subsequent data analysis.

*Drosophila* larvae navigate by using navigation strategies by means of processing spatial or temporal light information, defined in relation to the stimulus^64^. We measure spatial information processing by creating a directional light gradient, namely by presenting the light source from only one side of the behavioral plate (Fig. 1A). Through comparison of light information gathered from left and right eyes, larvae bias their heading direction away from the light source, either by steering or by making a turn. Accordingly, spatial navigation strategies are described as *mean run change* (the degree of steering within a run, biased away from the light source) and *turn direction* (the percentage of turns biased away from the light source). Navigational decision-making based on temporal cues can be assessed by using a temporal light gradient of recurring one-minute cycles, each encompassing a phase of linear light intensity increase and a corresponding phase of intensity decrease (Fig. 1B). Under this condition, larvae compare the light intensity change over time by sampling the environment through head-sweeps. Larvae make greater turns in size, turn more often and accept less head-sweeps when the environment becomes more unfavorable, which corresponds to the light intensity increase phase within the temporal setup. The opposite is true for the light intensity decrease phase, or the environment becoming more favorable for the animal. All temporal navigation strategies are measured distinctly for the two phases and consist of *turn size* (the degree of turn angle), *turn rate* (the average number of turns an animal makes per minute) and *head-sweep acceptance rate* (the percentage of accepted head-sweeps). These strategies are presented by a delta between the two light intensity phases. The overall navigation of larvae is demonstrated by *navigation index*, summarized by both spatial and temporal navigation strategies, and indicates directionality with respect to the light source where negative values represent navigation biased away.

Despite the complexity and level of detail in the described photo-behavioral programs, it remains unknown how the animals modulate navigational decisions in a circadian fashion. We first analyze navigation relevant behaviors as naïve responses at four different circadian times: dawn (CT0), midday (CT6), dusk (CT12) and midnight (CT 18). Intriguingly, none of the behaviors which are critically involved in navigation show circadian modulation in the absence of light stimulation. However, when a light source is introduced, we show that there is a strong modulation of the light-response during the course of the day with the largest difference being between dawn and midnight. We further find that each one of *pdf*, *per* and *clk* mutants show severe deficits in circadian modulated behaviors supporting that a functional molecular clock as well as proper neural signaling of pacemaker neurons is essential for maintaining circadian-modulated visually-guided navigational decision making.

## Results

Keeping animals in constant conditions is a commonly followed procedure applied prior to performing behavioral experiments, particularly important for circadian rhythm studies. Accordingly, placing animals in constant darkness (DD) enables the measurement of the direct effect of the light stimulus on the intrinsic molecular clock mechanism and averts the influence of the ‘masking effect’, which is the immediate adaptive response given to an environmental change^65^. Therefore, 2-day-old larvae are kept in DD for 2 days and the navigation of 3^rd^ instar foraging larvae is measured by using a computer-based tracking system as previously described^64^. This assay allows in-depth characterization of larval visually-guided navigation by dissecting it into distinct navigation strategies, dependent on either spatial or temporal information processing. Thus, our approach provides detailed insight about how larvae adjust their light avoidance behavior in accordance with circadian rhythm, and how this behavior is affected when the pacemaker mechanism is disrupted.

### In the absence of light stimulation, larval navigation strategies are not modulated by circadian rhythm

We first assessed the performance of navigation strategies in WT animals without presenting light stimulation, thus testing whether these behavioral parameters are intrinsically modulated by circadian rhythm. We measured larval navigation at four time-points, being subjective dawn (CT 0), midday (CT 6), dusk (CT 12) and midnight (CT 18). As described above, navigation strategies are defined in relation to the light source. Therefore, during data analysis of no-stimulus conditions, we eliminated directionality by including all heading directions, rather than considering the bias in heading direction as away from or towards the light source (see Methods).

**Figure 2:**
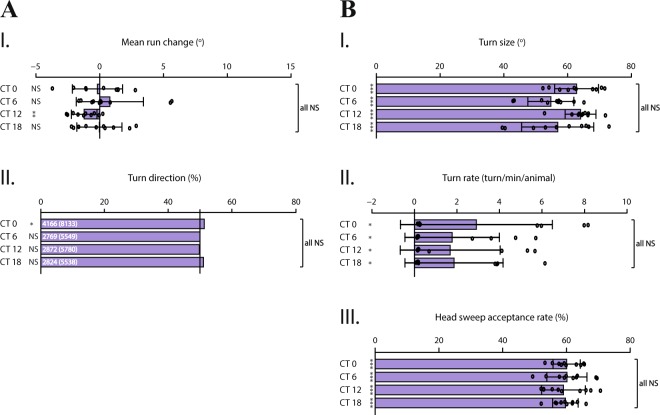
In the absence of light stimulation, larval navigation strategies are not modulated by circadian rhythm. All navigation strategies are analyzed according to no-stimulus conditions. Although larvae still perform distinct navigation strategies, without light stimulation, the heading direction is not biased and the efficiency of performance is not modulated in a circadian-dependent manner. **(A.I**,**A.II)** Spatial navigation strategies. **(A.I)** Although variable, the efficiency to steer within runs does not differ throughout the day (p > 0.05). **(A.II)** Under stimulus-naïve conditions, *turn direction* distinctly shows the percentage of turns made towards left in relation to all turns made, as the numbers indicated respectively on the bars. The direction of turns is not biased in the absence of light stimulus. **(B.I–B.III)** Temporal navigation strategies. Since no light stimulus is present, strategies are analyzed for all turns and head-sweeps made throughout the experiment period. **(B.I)** *Turn size* indicates the degree (angle) between the heading direction before and after a turn. Larvae adjust their turn size rather variably throughout the day. **(B.II)** *Turn rate* represents the average number of turns an animal makes in one minute. The frequency of turns at dawn shows a tendency to be higher compared to other tested time-points. **(B.III)** *Head-sweep acceptance rate* shows the percentage of accepted head-sweeps in relation to all head-sweeps made throughout the experiment. At all time-points, larvae accept head-sweep with approximately same rate. Data for each time-point are shown as means and the error bars indicate ± SEM. Circles indicate the means of individual experiments (n = 10). Statistical data can be found as Supplementary Table S1. *p < 0.05, **p < 0.01, ***p < 0.001, NS = not significant. Column statistics significance is indicated on the left side of each graph. All statistical comparisons between time-points are non-significant (p > 0.05), indicated as ‘all NS’ on the right side of each graph.

When no light stimulation is presented, we observed no time-dependent difference in the performance of both spatial and temporal navigation strategies (Fig. 2). The direction of turns is not biased and does not change along the course of the day (Fig. 2A.II). Likewise, temporal navigation strategies (Fig. 2B) indicate no circadian modulation, since none of the strategies are performed more or less prominently among indicated time-points. Although *mean run change* (Fig. 2A.I) and *turn size* (Fig. 2B.I) do not differ throughout the day (p > 0.05), it is noteworthy that these parameters are rather variable and larvae seem to have an increased turn frequency at dawn (Fig. 2B.II). Interestingly, the rate of accepting a head-sweep is approximately constant for all time-points and shows that more head-sweeps are accepted rather than rejected (Fig. 2B.III). This result might suggest that larvae accept head-sweeps by default and reject more often only when the environment is unfavorable (i.e. a head-sweep made towards light source). Taken together, our results indicate that in the absence of visual stimulation, navigation strategies are not subject to intrinsic circadian modulation.

### Both spatial and temporal light information processing are modulated by circadian rhythm

**Figure 3:**
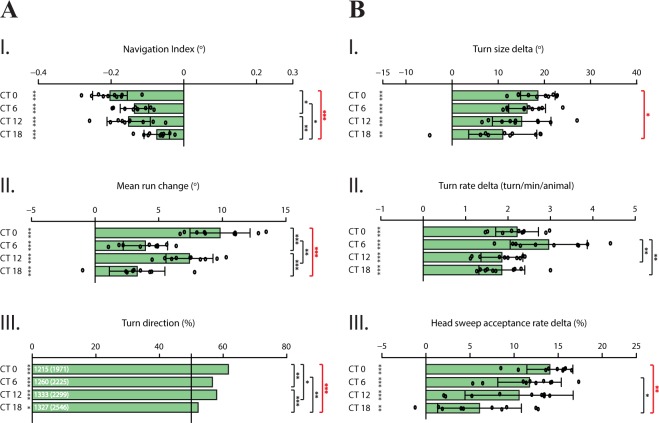
WT larvae avoid light more efficiently at dawn, especially compared to midnight. Distinct navigation strategies dependent on spatial and temporal information processing are subject to circadian modulation, leading to overall more efficient light avoidance behavior at dawn. For all navigation strategies, the largest modulation difference is observed between dawn and midnight. **(A.I–A.III)** Spatial navigation strategies. Negative values of *navigation index* indicate navigation away from the light source **(A.I)**. Larvae effectively avoid light at all tested time-points. Heading direction is biased by steering **(A.II)** and directing turns **(A.III)** away from the light source. The degree of steering away is higher at dawn and dusk, indicating more efficient avoidance behavior. Likewise, turn bias is conducted with the highest percentage at dawn. **(B.I–B.III)** Temporal navigation strategies. Single bars per time point plotted as *turn size delta*, *turn rate delta* and *head-sweep acceptance rate delta*, representing the difference between recorded values for light intensity increase and decrease phases. *Turn size delta* **(B.I)** and *head-sweep acceptance rate delta* (**B.III)** specifically point out to a diminished response given to temporal variation of visual input at midnight, especially compared to dawn. Interestingly, larvae turn more often at midday, although not divergent from values of dawn **(B.II)**. Data for each time-point are shown as means and the error bars indicate ± SEM. Circles indicate the means of individual experiments (n = 10). Statistical data can be found as Supplementary Table S2. *p < 0.05, **p < 0.01, ***p < 0.001, NS = not significant. Column statistics significance is indicated on the left side of each graph. All statistical comparisons between time-points are shown on the right side of each graph. To emphasize the consistent circadian modulation difference dawn and midnight, comparisons of these two time-points are shown in bold red.

In order to investigate circadian modulation of light avoidance behavior, we looked at WT larval navigation in the presence of both directional and temporal light gradient at chosen circadian times. Along the directional light gradient, larvae avoid light by preferentially navigating away from the light stimulus at all tested time-points, illustrated by negative values of *navigation index* (Fig. 3A.I). Larvae steer away more prominently at dawn and dusk (Fig. 3A.II), while the direction of turns is guided away relative to the light source most effectively at dawn (Fig. 3A.III). Together, higher values at specific time-points demonstrate a more potent bias, hence higher efficiency in performing these navigation strategies in order to avoid the light stimulus. Correspondingly, when a light gradient is presented over time, larvae are able to process temporal information. The difference (delta) between light intensity increase and decrease phases illustrated by temporal navigation strategies (Fig. 3B) expose the ability of larvae to adapt to environmental change induced by the temporal variation of visual input. Therefore, higher values of delta representation indicate higher potency in adaptation, presumably by regulating the sensitivity of visual input in a circadian-dependent manner. *Turn size delta* (Fig. 3B.I) does not show a prominent modulation difference throughout the day, except the performance of this navigation strategy at dawn in comparison to midnight indicates that larvae regulate the size of their turns more effectively at dawn, since the difference between the two light intensity phases is greatest at this time-point. Interestingly, larvae turn more often at midday, although not largely different from values at dawn (p > 0.05). This prominent ability to regulate the frequency of turns observed at midday, divergently from other navigation strategies, might indicate an influence of other drivers on this parameter, besides photophobicity (Fig. 3B.II). As the environment becomes more unfavorable during light intensity increase phase, larvae are expected to accept less head-sweeps while the opposite is true for light intensity decrease phase. Indeed, at all tested time-points, the rate of accepting a head-sweep is regulated by the light intensity change which is reflected by a higher difference between the two phases (Fig. 3B.III). Similar to *turn size delta*, regulating the rate of head-sweep acceptance is lower at midnight compared to other tested time-points, suggesting a diminished response towards the temporal change of visual input.

Taken together, these findings suggest that distinct navigation strategies dependent on both spatial and temporal information processing are subject to circadian modulation, leading to more efficient light avoidance performance especially at dawn. The opposite is true for midnight; all navigation strategies are least effectively executed at this time-point. At midday and dusk, larvae perform navigation strategies with interchangeable efficiency, presumably due to either circadian modulation difference between these two time-points being not strong enough to be reflected by light avoidance behavior, or due to circadian modulation being established differently on spatial and temporal navigation strategies. The time-dependent difference in navigation might be attributable to higher sensitivity to light stimulus, specifically at dawn^66^, presumably through the impact of circadian signaling transmitted via clock neurons on the visual central circuitry, as recently described^67^.

Collectively, the results obtained from stimulus-naïve and light-stimulus conditions indicate that circadian modulation of navigation strategies apparent in the presence of light stimulation likely reveals the rhythmicity of light information processing itself, rather than a default variation of larval behavior during the day. The largest modulation difference was observed between dawn and midnight. Therefore, we proceeded further testing specifically at these two time-points. To confirm that the observed difference of light information processing between dawn and midnight is a specific effect of the biological clock, we next used animals deficient for genes previously described to be important in maintaining the daily rhythmic activity.

### PDF neuropeptide is required for circadian modulation of photophobic navigation in DD conditions

Although neurons that constitute the circadian pacemaker circuitry are known, individual roles of these neurons and the signal transmitting circadian information within discrete behaviors are yet to be established. PDF-LaNs are the main circadian pacemaker neurons^68^ and the only known neurons to transmit information via PDF neuropeptide signaling. In adult flies, PDF neuropeptide is expressed by ventral lateral neurons (LN_v_s)^69^, which are necessary to establish locomotor rhythms^70,71^, specifically for inducing the morning activity peak^72^. In LD conditions, *pdf* mutants retain activity rhythms, although PDF is necessary to produce the morning activity peak and to phase the evening activity peak^72,73^. When mutants are switched to DD, activity rhythms are lost in the absence of PDF signaling^73^. Therefore, in adult flies PDF is required to transmit the information that synchronizes the phase and the amplitude of circadian rhythms among pacemaker neurons and to maintain rhythmicity under constant conditions^74^.

**Figure 4:**
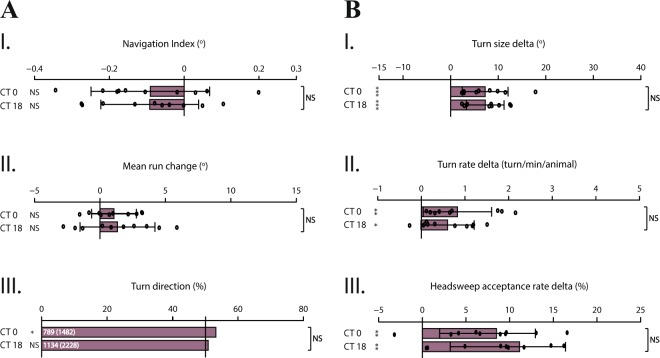
*pdf  *^01^ larvae exhibit disrupted circadian modulation and dampened light avoidance behavior especially at dawn. *pdf  *^01^ mutant larvae are able to perform navigation strategies dependent on both types of information processing. However, no substantial difference in light avoidance behavior is observed between dawn and midnight, indicating absence of circadian modulation. More precisely, in accordance with the role of PDF in establishing the morning activity peak in adult flies, higher sensitivity to light stimulation at dawn coupled with more efficient light avoidance is not observed. Larvae perform this behavior uniformly. **(A.I–A.III)** Spatial navigation strategies. **(B.I–B.III)** Temporal navigation strategies. Data for each time-point are shown as means and the error bars indicate ± SEM. Circles indicate the means of individual experiments (n = 10). Statistical data can be found as Supplementary Table S3. *p < 0.05, **p < 0.01, ***p < 0.001, NS = not significant. Column statistics significance is indicated on the left side of each graph. All statistical comparisons between time-points are indicated on the right side of each graph.

We asked whether PDF neuropeptide is involved in transmitting the information of rhythmic activity in photophobic navigation of larvae kept in DD conditions. For this purpose, we tested light avoidance behavior of *pdf* mutants, which were shown to exhibit defective activity rhythms while not comporting developmental defects^16,75^. Our results indicate that, compared to WT, the efficiency of *pdf  *^01^ larvae in performing both spatial and temporal navigation strategies is considerably dampened, especially at dawn, as summarized by the overall navigation index (Fig. 4A.I). Notably, the heading direction of larvae seems not to be biased, denoted by rather minor values of *mean run change* and *turn direction* (Fig. 4A.II,III). On the other hand, *pdf  *^01^ larvae seem to be able to process temporal light information effectively, regulating turns by adjusting the size and rate, and head-sweeps by accepting or rejecting (Fig. 4B) as a behavioral response to temporal light intensity variation. Overall, unlike WT larvae, no difference in light avoidance behavior between dawn and midnight is observed, which indicates that circadian modulation of light information processing is lost when PDF signaling is disrupted. Therefore, PDF neuropeptide seems to be necessary to maintain activity rhythms of larvae in constant conditions, as in adult flies.

### Larvae with mutated molecular clock show defects in circadian modulation of light avoidance behavior

**Figure 5:**
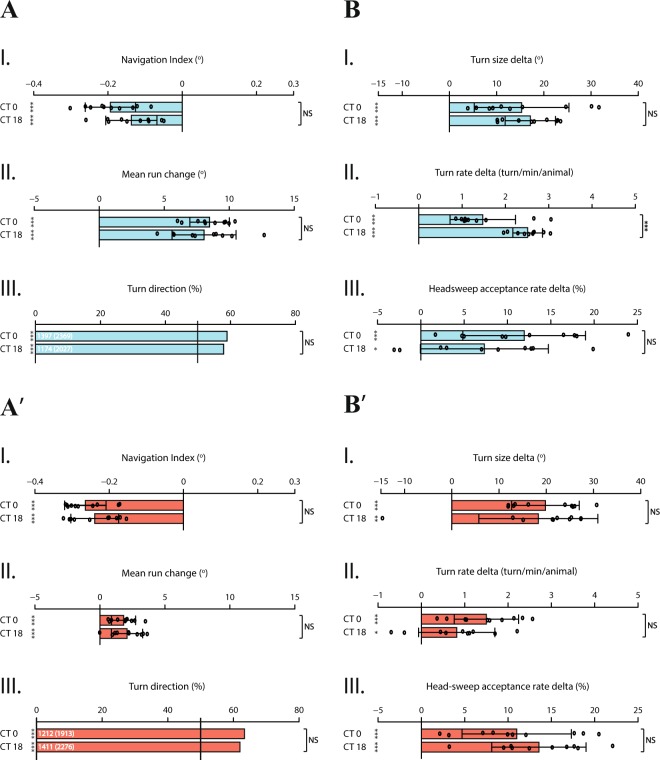
A properly functioning molecular clock mechanism is necessary for circadian modulation of light avoidance behavior. Larvae with mutated clock mechanism, *per*^01^ and *clk*^*Jrk*^ are tested for their light avoidance behavior. Both mutants are still able to perform spatial and temporal navigation strategies through processing the corresponding information. However, since the clock mechanism is disrupted, circadian modulation is abolished for both types of navigation strategies. **(A,B)** *per*^01^ larvae. **(A.I–A.III)** Spatial navigation strategies. **(B.I–B.III.)** Temporal navigation strategies. **(A’,B’)** *clk*^*Jrk*^ larvae. **(A’.I–A’.III)** Spatial navigation strategies. **(B’.I–B’.III)** Temporal navigation strategies. Data for each time-point are shown as means and the error bars indicate ± SEM. Circles indicate the means of individual experiments (n = 10). Statistical data can be found as Supplementary Table S4. *p < 0.05, **p < 0.01, ***p < 0.001, NS = not significant. Column statistics significance is indicated on the left side of each graph. All statistical comparisons between time-points are indicated on the right side of each graph.

Larval clock neurons are distinguishable from other neurons by their rhythmic activity of core clock components; *period* (*per*), *timeless* (*tim*), *cycle* (*cyc*) and *clock* (*clk*)^48^. In order to substantiate circadian modulation of larval light avoidance behavior, we asked how this modulation would be affected when the molecular clock mechanism was disrupted. We therefore tested *per* null mutant larvae (*per*^01^)^76^ as well as *clk*^*Jrk*^ larvae for light avoidance behavior at dawn and midnight. Circadian oscillation of transcriptional and translational products of the *per* gene results from and contributes to molecular circadian rhythms, and moreover, these products are translated into behavioral rhythms^77,78^. Under free-running conditions, mutations in *per* gene locus have a direct effect on rhythmicity; missense mutations lengthen (*per*^L^) or shorten (*per*^S^) the circadian period, while null mutations lead to complete loss of rhythmicity^76,79–81^. Similarly, *clk*^*Jrk*^ larvae have completely arrhythmic locomotion in DD conditions and encode for a truncated CLK protein leading to extremely low and non-cycling PER and TIM levels, due to reduced transcription of respective genes^32,33^. Our data show that both mutant larvae are still able to perform light avoidance behavior through spatial and temporal navigation strategies, demonstrated by robust values of *navigation index* (p < 0.05) (Fig. 5A.I,A’.I). Larvae bias their heading direction away from the light source both by steering (Fig. 5A.II,A’.II) and by adjusting turn direction (Fig. 5A.III,A’.III). Notably, although still biased, *clk*^*Jrk*^ larvae display dampened performance of steering within runs (Fig. 5A’.II). However, circadian modulation that attunes the light information processing observed in WT larvae is no longer present; mutant larvae bias their heading direction with the same efficiency at dawn and midnight. Likewise, mutant larvae regulate the degree of turns as a response to light stimulus (Fig. 5B.I,B.I’) without any distinction in performance between tested time-points. Additionally, as shown by *turn rate delta*, *per*^01^ larvae turn more often at midnight (Fig. 5B.II), which is the opposite of how circadian modulation impacts the performance of this navigation strategy specifically in WT larvae. Interestingly, *per*^01^ and *clk*^*Jrk*^ larvae display an opposite trend in performing temporal navigation strategies (Fig. 5B,B’); as WT larvae, *clk* mutants have an inclination to regulate their turns and head-sweeps in response to temporal variation of visual input more prominently at dawn, while on the contrary, per mutants show this bent at midnight. Tendencies in performing these temporal navigation strategies are presumably due to loss of circadian modulation, hence possible arrhythmic behavior of individuals, as shown by previous studies^32,76,80^.

## Discussion

For efficient photo-navigation, *Drosophila* larvae process both spatial and temporal cues^64^. Through comparison of light information collected by left and right eyes, spatial navigation strategies, as described for *taxis*^82^, presuppose a direct behavioral response to the stimulus intensity and directional orientation bias. Conversely, temporal information processing, corresponding to *kinesis*^82^, involve temporal comparison of stimulus intensity and adjustment of turns, as in size and rate, and head-sweeps, as in acceptance or rejection. Previous studies revealed that circadian rhythm regulates the light-dark preference of *Drosophila* larvae, measured through a light-dark preference assay^66,83^ in which only half of the experimental plate is illuminated. Although this assay allows robust measurements of larval light avoidance, it does not expose distinct navigation strategies underlying the avoidance behavior. Moreover, this assay favors the measurement of temporal information processing, since spatial comparison is only limited to the mid-line that separates light and dark sides of the experimental plate. Our data adds a level of understanding of the daily rhythmicity of photophobic navigation in fruit fly larvae by disintegrating this behavior into distinct navigation strategies. Furthermore, we show that the described rhythmic variation is dependent on light information processing itself, absent in no-stimulus conditions.

In stimulus-naïve conditions, navigation strategies dependent on either spatial or temporal information processing are still performed for navigation although without any bias, presumably for food seeking or exploring the environment. Nonetheless, these navigation strategies are not subject to circadian modulation since larval navigation does not show any time-dependent alteration throughout the day. It could be argued that the variable values obtained for *mean run change* (Fig. 2A.I) and *turn size* (Fig. 2B.I) might be due to larval intrinsic activity rhythms. Adult flies anticipate the light-dark transition phases through their biological clock and increase their activity according to this anticipation^10^. It appears possible that the same activity rhythm pattern might also be true for larvae. However, since these two navigation strategies were the only behavioral parameters to suggest this pattern, further experiments which specifically investigate activity rhythms should be conducted. Additionally, the tendency for higher turn frequency at dawn illustrated by *turn rate* (Fig. 2B.II) could be indicative of increased activity at this time. Nevertheless, this tendency alone is not sufficient to support that turn rate would represent larval intrinsic activity modulation by circadian rhythm. Therefore, we conclude that the tendencies observed for these temporal navigation strategies performed in the absence of light stimulation reflect behavioral noise rather than circadian rhythm-induced patterns.

When a light stimulus is presented, larvae avoid light with high efficiency especially at dawn, demonstrated by a stronger bias in the heading direction away from the light source (Fig. 3A.II,A.III). However, it is noteworthy that a tendency to perform spatial navigation strategies more effectively at dusk is observed as well, while this performance was less prominent at midday and midnight. On the other hand, a trend for performing temporal navigation strategies comparable to dawn was observed at midday. For both types of navigation strategies, the efficiency in performance was dampened at midnight. Collectively, an intensive response to both spatial and temporal visual input given particularly at dawn might be due to increased sensitivity to light stimulus. Circadian gating of light information could intensify or diminish the behavioral response. Since the least prominent avoidance performance of all navigation strategies are observed at midnight, the opposite might be true for this time-point. Presumably due to least expectancy of environmental threats such as predators, desiccation and DNA-damage by exposure to ultraviolet light, larvae might be rendered relatively less sensitive to visual stimulus, and rather prioritize other sensory modalities such as chemical-sensing, in accord with circadian rhythms facilitating adaptation to environmental conditions^49^. Another possible explanation for the strong modulation difference observed between dawn and midnight might be the resting state of larvae. Under 12 h LD conditions, adult flies restrict their activity to subjective day and display sleep-like resting behavior, described as resting states during which flies are less responsive to sensory stimuli^84,85^. Like their adult counterparts, larvae sleep as well, defined by rapidly reversible quiescent states, which is crucial for their development^86^. Although a clear connection between time of the day and resting behavior is currently lacking in larvae, during nighttime animals might be rendered relatively less sensitive to sensory stimuli, as their adult counterpart. Although strong light stimulation disturbs the sleep state^86^, which is supported by our results showing that larvae use navigation strategies to avoid light throughout the day, circadian modulation presumably defines the animal’s sensitivity to light stimulation. As a result, navigation strategies used for light avoidance are seemingly performed more prominently at dawn, likely due to higher arousal by the stimulus, and conversely, less prominently at midnight. Other tested time-points, midday and dusk, showed variable efficiency in performing avoidance behavior. As illustrated by substantial differences between midday and dusk in conducting *mean run change* and *turn rate delta*, it could be argued that a tendency to perform spatial navigation strategies (Fig. 3A) more efficiently at dusk is observed while temporal navigation strategies (Fig. 3B) tend to be performed more prominently at midday. This interchangeable efficiency in performance might be explained by the modulatory effect of circadian rhythm being established distinctly on spatial and temporal information processing, leading to variant efficiency in performance. However, this conclusion can only be reached with further elucidation of how this modulation impacts the neural circuitry which regulates larval navigation.

With the exception of DNs, the pacemaker circuitry is a direct synaptic target of photoreceptors (PR)^67^, exposing the central role of light sensing in circadian entrainment. Correspondingly, the two separate PR pathways, delineated by blue-tuned Rhodopsin 5 (Rh5)- and green-tuned Rhodopsin 6 (Rh6)-expressing PRs, show segregated but also overlapping function in light avoidance behavior and circadian entrainment^64,68^. Larval main pacemaker neurons, PDF-LaNs, are the only shared downstream targets of Rh5- and Rh6-PRs^67^, in compliance with the finding that either PR-subtype is sufficient to entrain the molecular clock^68^. On the contrary, both PR-subtypes are necessary for efficient navigation due to their distinct roles; Rh6-PRs are required for temporal information processing whereas Rh5-PRs seem to be predominantly necessary for spatial information processing but also for the integration of both types of light information for downstream transmission^64^. Besides PDF-LaNs, Rh5-PRs also project onto other visual projection neurons, being postero-ventro-lateral neuron 09 (PVL09), projection optic lobe pioneer neurons (pOLP), fifth-LaN and non-clock LaNs, whereas Rh6-PRs only project onto local optic lobe pioneer neurons (lOLP) which are visual interneurons characterized by cholinergic and respectively glutamatergic neurotransmitters, presumably of opposed valence (excitatory/inhibitory)^87^. The lOLPs present reciprocal connections with each other and project onto other visual interneurons, creating circuit modulatory motifs^67^. Interestingly, the lOLPs also create such presumed excitatory-inhibitory motifs with PDF-LaNs, possibly tuning their activity according to the received temporal light information. Thus, lOLPs are likely responsible for temporal comparison of light information^88^ for further attuning of the entire visual system. Since spatial and temporal information processing are segregated through distinct PR pathways, it could also be possible that circadian modulation of visual interneurons is distinctly established on corresponding neuronal circuit components. More specifically, it could be envisaged that visual interneurons regulating temporal information processing are first subject to circadian modulation, conveying further into the circuit through their characteristic modulatory motifs. Alternatively, circadian modulation might be simultaneously established on projection visual interneurons and integrated into downstream connections. Elucidating the connectivity characterizing PDF signaling, additional circadian modulators and the expression of corresponding receptors would shed light on the neurons directly targeted by the pacemaker system for circadian tuning of light information processing, as well as other sensory modalities.

Considering the distinct involvement of Rh5- and Rh6-PR pathways in mediating spatial and respectively temporal information processing, we contemplated that PDF signaling could play a role in the performance of these navigation strategies, since PDF-LaNs receive direct synaptic input from both PR-subtypes. Indeed, mutants deficient in PDF signaling present not only an abolished circadian modulation, but also a considerably dampened performance of navigation strategies compared to WT larvae. This dampening is especially noted for dawn, consistent with the involvement of PDF neuropeptide in adult flies, establishing the morning activity peak in LD conditions and in maintaining rhythmicity in DD conditions. The underlying cause for dampened photo-navigation observed in larvae might be disrupted communication between PDF-LaNs and downstream neurons responsible for larval navigation. However, it remains yet to be determined whether light information is directly conveyed via PDF-LaNs or via additional connections made with downstream visual interneurons. Further dissection into the system is necessary in order to characterize the neuronal targets of PDF-LaNs.

*Pdf  *^01^ larvae have disrupted PDF-neuropeptide signaling, which is necessary for synchronizing larval pacemaker circuitry^74^. Nonetheless, these mutants have an intact molecular clock mechanism which still undergoes circadian oscillations. Divergently, we also tested *per*^01^ and *clk*^*Jrk*^ larvae in order to investigate the impact of a disrupted molecular clock mechanism on circadian modulation of light avoidance behavior. Given that cycling of *per* mRNA and PER is essential for constituting rhythmic behavior, *per* null mutants show a prominent phenotype of circadian modulation loss. At the molecular level, CYC and CLK might still act as activators for other genes, however, *per* gene products as the main clock component which dictate rhythmicity is lost. Presumably, the only rhythmicity indicator in *per*^01^ larvae is light-dependent degradation of TIM, induced by CRY. However, under free-running (DD) conditions TIM cannot be degraded and the timing of PER/TIM nuclear translocation defines the rhythmicity of the animal^17^. Thus, *per*^01^ larvae have no molecular oscillations which would indicate circadian rhythmicity in DD, in accord with our findings demonstrating disrupted circadian modulation of navigation strategies. Likewise, *clk*^*Jrk*^ larvae are able to regulate heading direction, turns and head-sweeps in relation to light stimulus, however, this avoidance behavior is not subject to circadian modulation due to disrupted molecular clock mechanism. Given that CLK is a component of the heterodimer that acts as an activator of *per* and *tim* genes, protein levels encoded by these genes are extremely low and non-cycling in *clk* mutants^32,33^. Therefore, defects observed in circadian modulation of light avoidance behavior similar to *per*^01^ larvae appears to be coherent. Nevertheless, PER and CLK constitute the negative and respectively the positive components of transcriptional feedback loop, which means that at the molecular level these mutants might entail disruption of the molecular clock mechanism in different aspects, consistent with divergent roles described for these mutants^30,52^. Notably, these mutants display tendencies in opposite directions in performing temporal navigation strategies (Fig. 5.B,B’). Considering loss of circadian modulation and previous studies revealing arrhythmic behavior of both mutants, we interpret these tendencies as possible indication of arrhythmicity. Overall, results obtained from larvae with mutated clock components demonstrate that alterations in the signaling and the molecular integrity of the clock circuitry lead to loss of circadian modulation of photophobic navigation, substantiating the modulation observed in WT larvae, along with no intrinsic modulation of distinct navigation strategies observed under stimulus-naïve conditions.

## Methods

### Fly strains

*Drosophila melanogaster* flies were grown on cornmeal medium at 25 °C under a 12 h light-dark cycle. Fly lines used for behavior experiments were: wild-type Canton-S, *pdf  *^01^ (Bloomington 26654), *per*^01^ and *clk*^*Jrk*^ (gift from Dennis Pauls and Charlotte Helfrich-Förster).

### Preparation of behavior experiments

All experiments were set, performed and analyzed as detailed previously^64^. Adult flies were allowed to lay eggs for 24 hours under 12 h light-dark (LD) conditions. 2-day-old larvae entrained to LD conditions were placed into constant darkness (DD) for 2 days. Behavior experiments were performed using 3^rd^ instar foraging larvae (4-day-old). We took as premise that after solely two days in free-running state, the internal clock faithfully reflects the circadian times the animals were entrained to. Therefore, experimental time-points are indicated by circadian time (CT), respectively as CT 0, CT 6, CT 12 and CT 18. For each CT, the experiment was designed to start 1 hour before and to end 1 hour after the given time point (i.e. CT 5 - CT 7 for CT 6 experiments). For each time-point, experiments were repeated ten times. Each experiment included thirty larvae collected from the food and washed twice with tap water at room temperature. A behavioral plate was prepared by using a 24.5 × 24.5 cm petri dish (BD Falcon BioDishXL, BD Biosciences) with an aluminum plate on the bottom to create contrast, covered homogenously with 2,5% agarose (Agarose Standard, Roth). After the agarose cooled down to room temperature, larvae were placed in the middle of the plate for behavioral recording. All experiments were performed under red-light illumination.

### Larval behavior tracking

The behavioral plate was placed in a box in darkness, illuminated evenly from each side by four strings of red-light LEDs (623 nm, Conrad). To record larval navigation, a computer-based tracking system was used as previously described^61,63^. Throughout the experiment, larvae were recorded by a camera (acA2500-14 gm, Basler AG, Germany) equipped with a lens (Fujinon HF12.5HA-1B 12.5 mm/1.4, Fujifilm, Switzerland) and a red-light bandpass filter (BP635, Midwest Optical Systems, USA), at a rate of 13 frames per second. The camera was placed 45 cm above the center of the agarose-filled behavioral plate. Larvae were allowed to move freely while being recorded for 11 minutes per experiment, out of which the first minute was considered necessary for larval acclimatization to the environment and therefore excluded during data analysis. For image acquisition, a custom-made LabView software was used and the data was analyzed by the MAGAT Analyzer^61,63^. Follow-up analysis and data visualization were performed using MATLAB and R Studio.

### Visual stimulation

Two different setups were used to measure distinct navigation strategies, both designed to present light stimulation within the overall spectrum defined as UV-A to green^89–91^, covering the sensitivity range of the two different larval photoreceptor subtypes. To measure spatial navigation strategies, a directional light gradient was created by a projector (EX7200 Multimedia Projector, EPSON) equipped with a bandpass filter (335–610 nm, BG40, Thorlabs), illuminating the behavioral plate from one side. The projector was placed with a 40° incline, 26 cm height and 38 cm away from the behavioral plate center where larvae were placed. Maximum light intensity was 4331 μW/cm^2^ with two maximum intensity peaks at 71.6 μW/cm^2^, 438 nm, half-width: 9 nm, and respectively at 47.9 μW/cm^2^, 549 nm, half-width: 10 nm. To measure temporal navigation strategies, instead of a projector, the behavioral plate was illuminated by blue and green LEDs (PT-120, Luminus, Billerica, MA, USA) placed 45 cm above and perpendicular to the surface of the behavioral plate. Maximum light intensity was 378 μW/cm^2^ (first intensity peak at 11.9 μW/cm^2^, 455 nm, half-width: 9 nm; second intensity peak at 3.7 μW/cm^2^, 522 nm, half-width: 14 nm). To create a temporal light gradient, the light intensity of LEDs was modified by linearly increasing and decreasing phases controlled by an Arduino-based customized script. Each progressive phase was set to last 25.5 s and was followed by a constant phase of 4.5 s at maximum (after a step of light increase) and respectively at minimum light intensity (after a phase of decrease). One temporal light cycle was therefore completed in 1 minute and was repeated 10 times for each experiment. The rate of light intensity change was approximately 1.5 μW/cm^2^ every 100 ms. Only larval behavior during linear light intensity increase and decrease was taken into account for subsequent data analysis.

### Navigational parameters

Navigation strategies were defined and analyzed as previously described^64^. Larvae were detected by the tracking system based on their shape and size and each individual animal trajectory was converted into a track, subsequently segmented into runs and turns by the customized MAGAT Analyzer software^61^. Briefly, runs were defined as forward movements in which the body and head are aligned. When a run stops, the larva makes a head-sweep by casting the head towards one side, abolishing the head-body alignment. The head-sweep can either be accepted or rejected. Head-sweeps were defined as rejected if followed by another head-sweep. Once a head-sweep is accepted, a turn is made towards the direction of the head-sweep and a new run is initialized. Hence, a turn can be defined as a reorientation event as a result of an accepted head-sweep.

The *navigation index* represents the overall navigation performance of larvae, calculated by the mean velocity along x-axis divided by the mean run speed in all directions^64^. Negative values indicate navigation away from the light source. To analyze the navigation strategies, a navigational compass characterizing the heading direction of larvae was used. The compass was divided into four 90° segments. Larval navigation with respect to the light source was identified as: 0° towards the light source, 180° away from the light source, ±90° perpendicular to the light source. Navigation strategies measured in the presence of directional light gradient, termed as spatial navigation strategies, were defined and analyzed as: *mean run change*, the degree of steering biased away from the light source within a run, only when larvae were heading in the direction of ±90°; *turn direction*, the percentage of turns biased away from the light source, in relation to all turns made, only when larvae were heading in the direction of ±90°.

Correspondingly, temporal navigation strategies performed in the presence of temporal light gradient cycles were analyzed separately for light intensity increase and decrease phases, defined as: *turn size*, the difference in terms of degree between the heading direction before and the heading direction after a turn; *turn rate*, the average number of turns made by the average number of animals in one minute; *head-sweep acceptance rate*, the percentage of head-sweeps that have been accepted rather than rejected.

All graphs were plotted as bars per time-point (CT), indicating an overall mean derived from individual means of each repetition. For *turn size, turn rate* and *head-sweep acceptance rate*, a single bar was plotted representing the difference (delta) between the means of light intensity increase and decrease phases (as indicated on figure title) per time-point, obtained by subtracting values of former from the latter.

For experiments performed in the absence of light stimulation, navigation strategies were defined as described above, except that directionality was eliminated during data analysis by including all runs, turns and head-sweeps. Since no bias would be expected, for *mean run change* and *turn direction*, instead of selecting only larvae heading in the direction of ±90° (perpendicular to the light source), all heading directions were taken into account. Turns made towards left in relation to all turns made were calculated for *turn direction*. For *turn size* and *turn rate*, an overall mean derived from individual means of each experimental repetition was indicated instead of the difference (delta) between light intensity increase and decrease phases. *Navigation index* was not presented for no-stimulus conditions since it is normally calculated as the navigation along the x-axis with respect to the light source.

### Statistical analysis

Statistical analyses were defined and conducted as previously described^64^. Data were plotted in column bars as mean with SEM. As each experiment was repeated 10 times, the means of individual experiments were illustrated by circles. Statistic functions in MATLAB (t-test) and RStudio (binom.test, fisher.test, aov and glht (multcomp)) were employed as follows. A two-tailed one sample t-test was performed to test the overall mean of each time-point (CT) against chance for each navigation strategy, except for *turn direction* where the probability was tested using a two-tailed exact binomial test. A one-way ANOVA followed by a Dunnett’s test was applied to test for statistical comparison between different CTs for *navigation index*, *mean run change*, *turn size*, *turn rate* and *head-sweep acceptance rate*. A two-tailed Fisher’s exact test was used for *turn direction* to test for statistical comparison between different CTs, and also to test for the distribution of accepted and rejected head-sweeps between light intensity increase and decrease phases. For *turn direction*, which presents a binary choice, numbers of events were indicated on bars as: number of turns biased away from the light source, total number of turns in brackets; and respectively, number of accepted head-sweeps, total number of head-sweeps made in brackets. Rejection of null hypothesis (being the same or chance): *p ≤ 0.05, **p ≤ 0.01, ***p ≤ 0.001. In case of multiple comparisons, p-values were adjusted by the Benjamini Hochberg procedure.

## Supplementary information

Supplementary information.

## Data Availability

The datasets generated during and/or analyzed during the current study are available from the corresponding author upon reasonable request.
